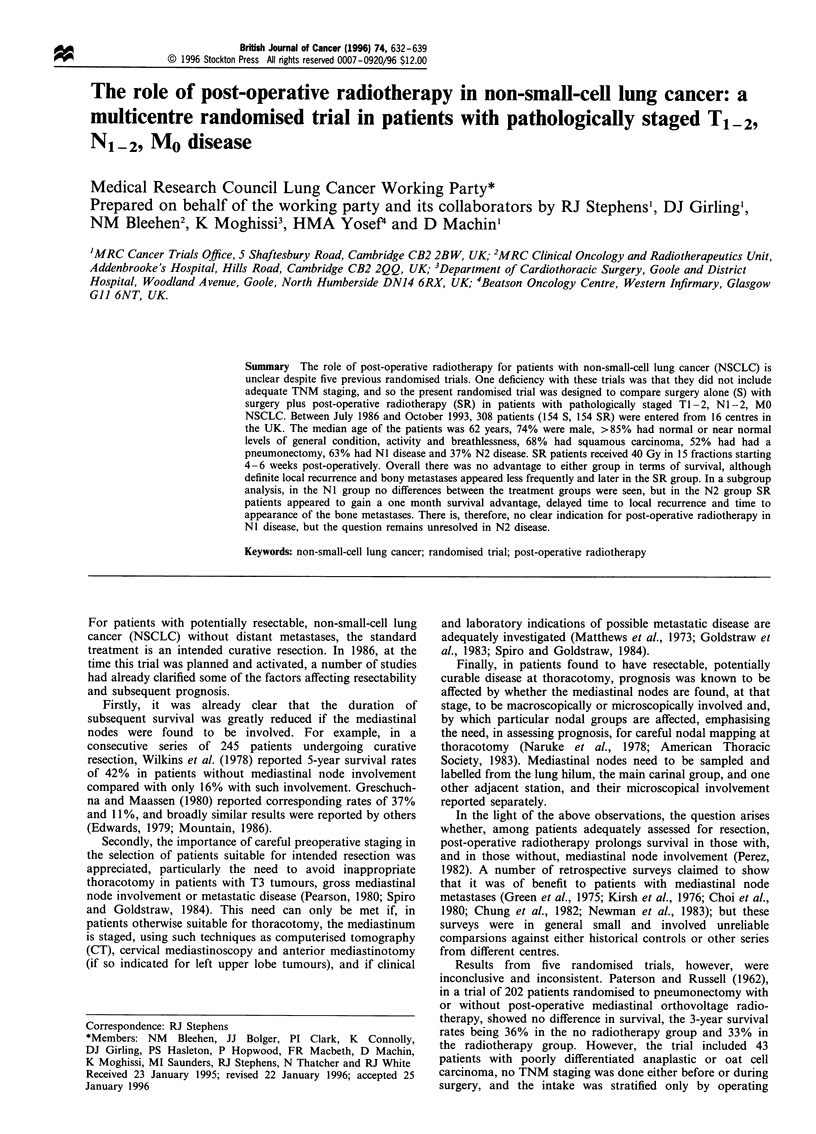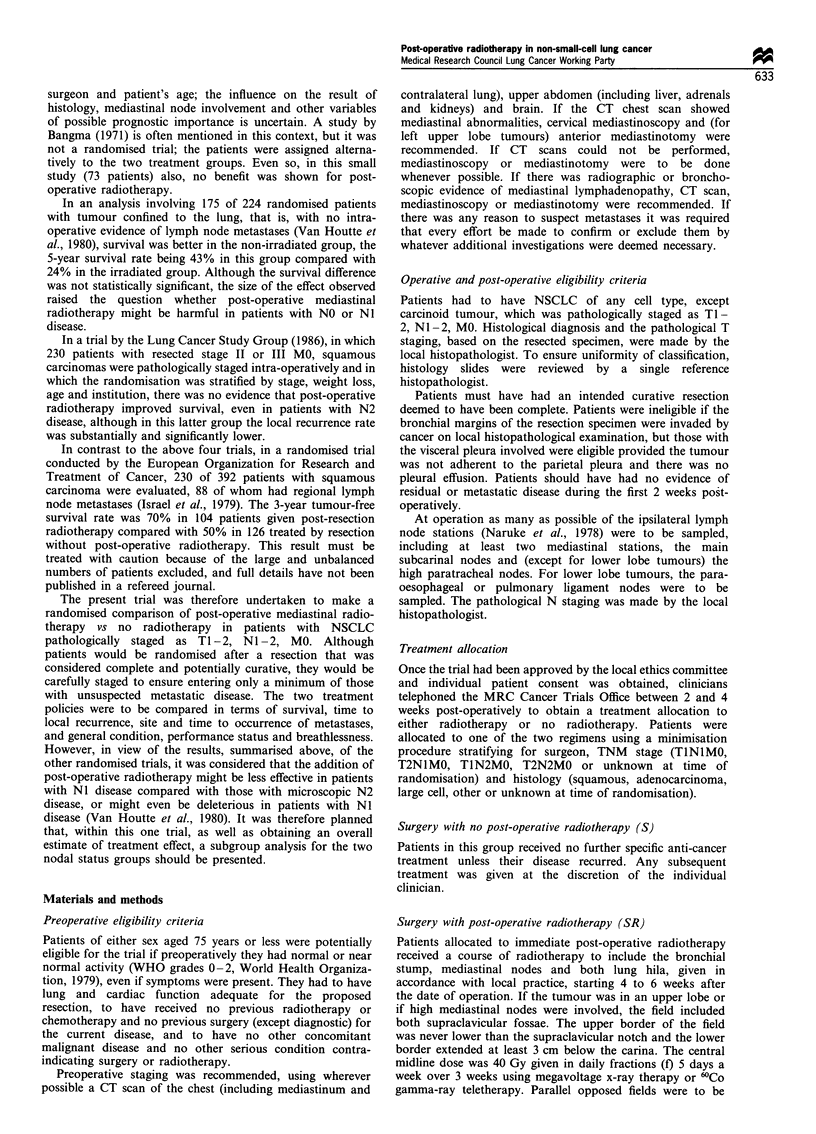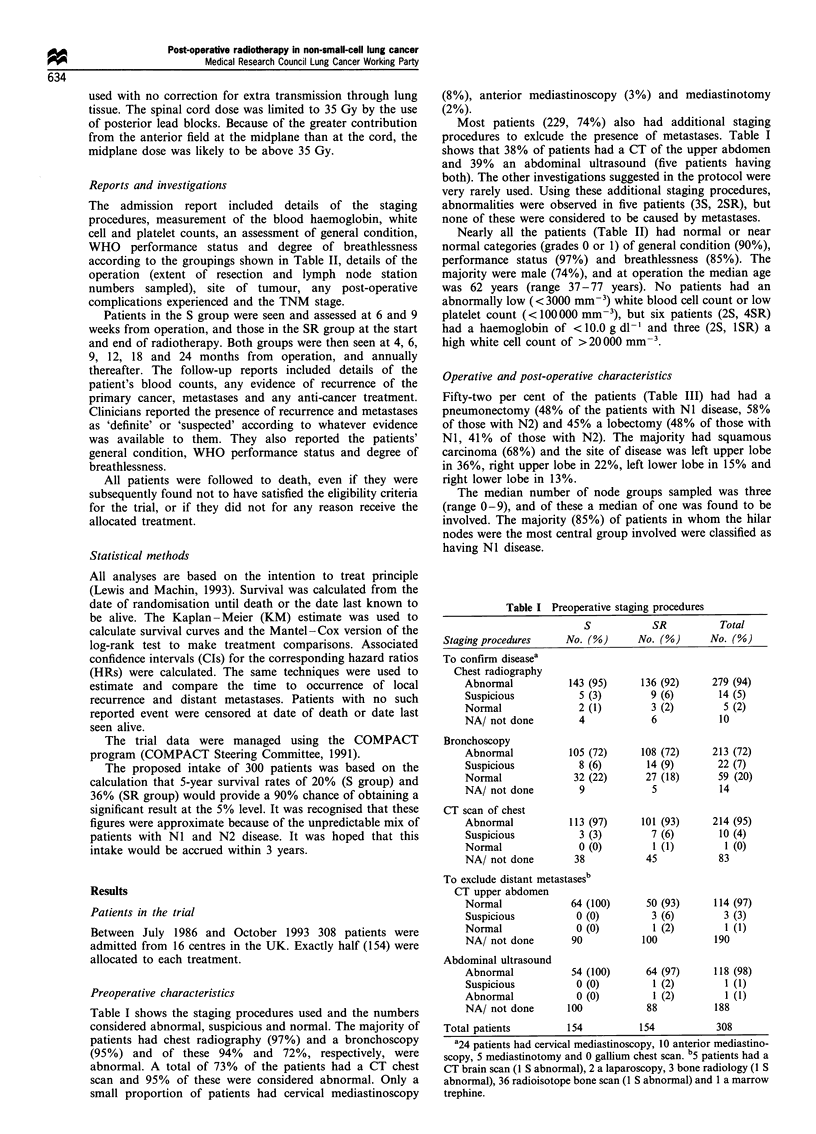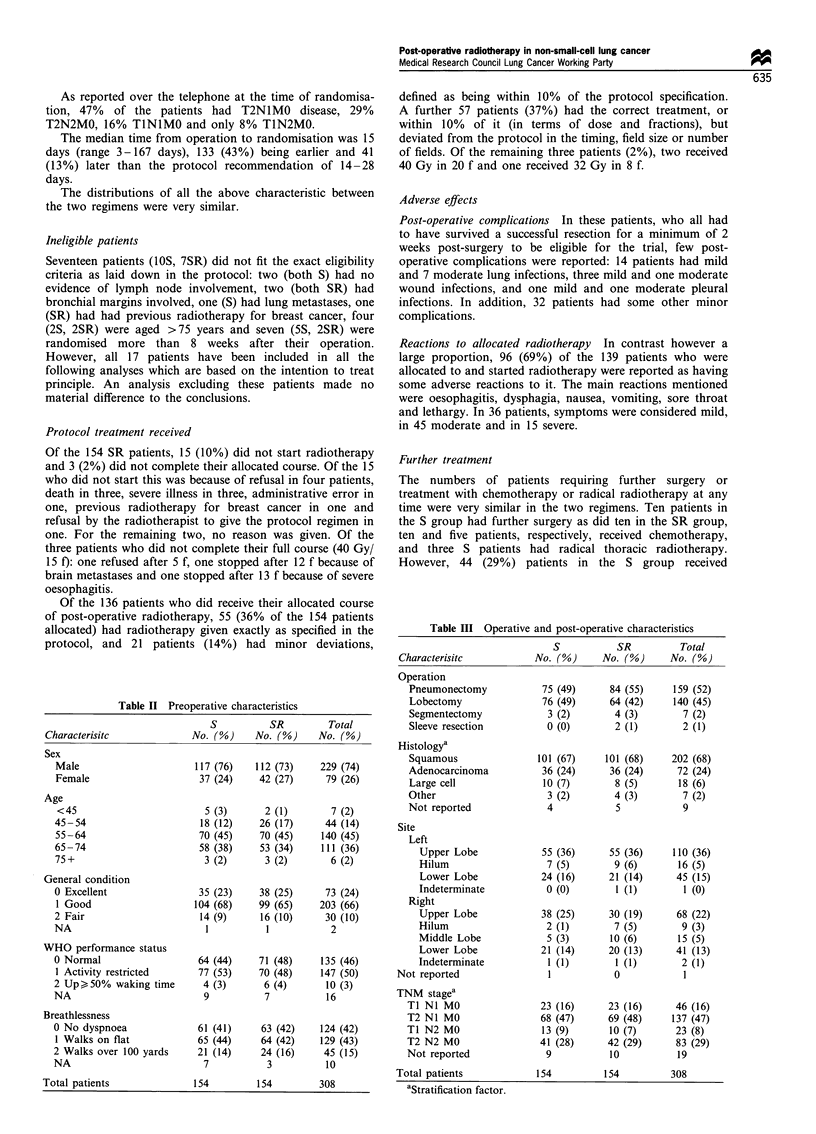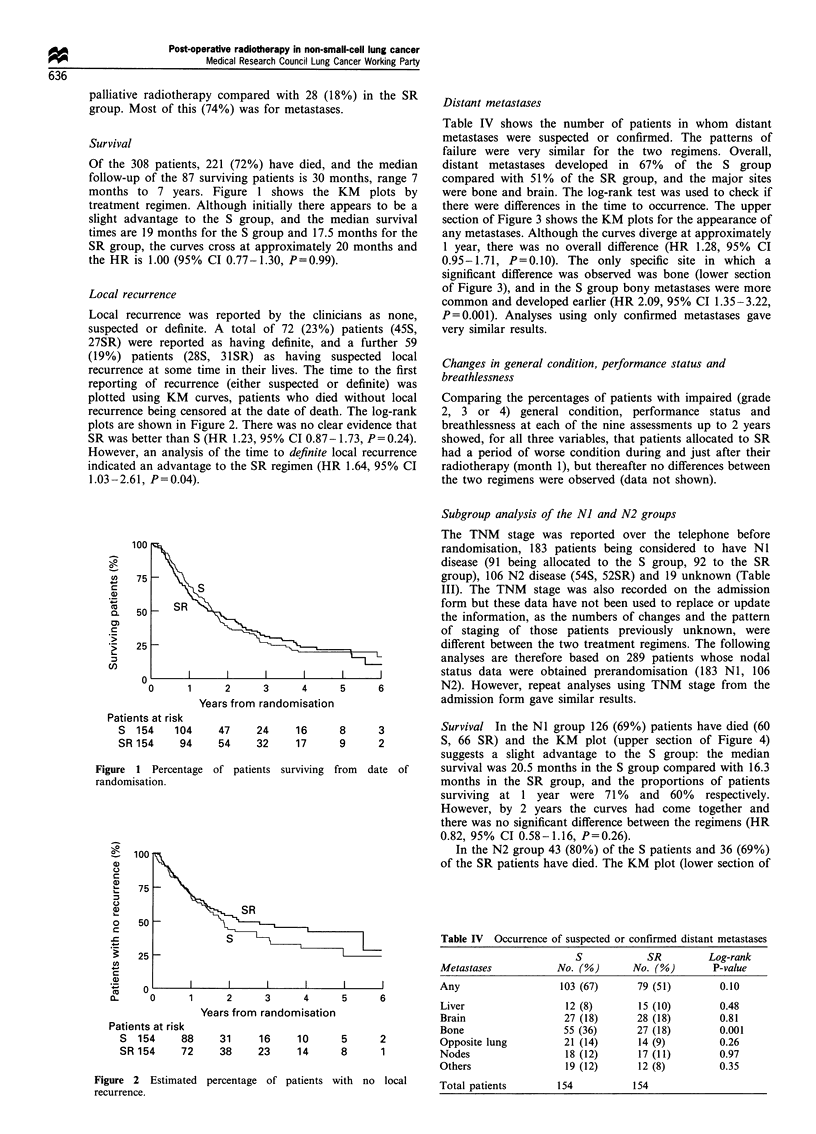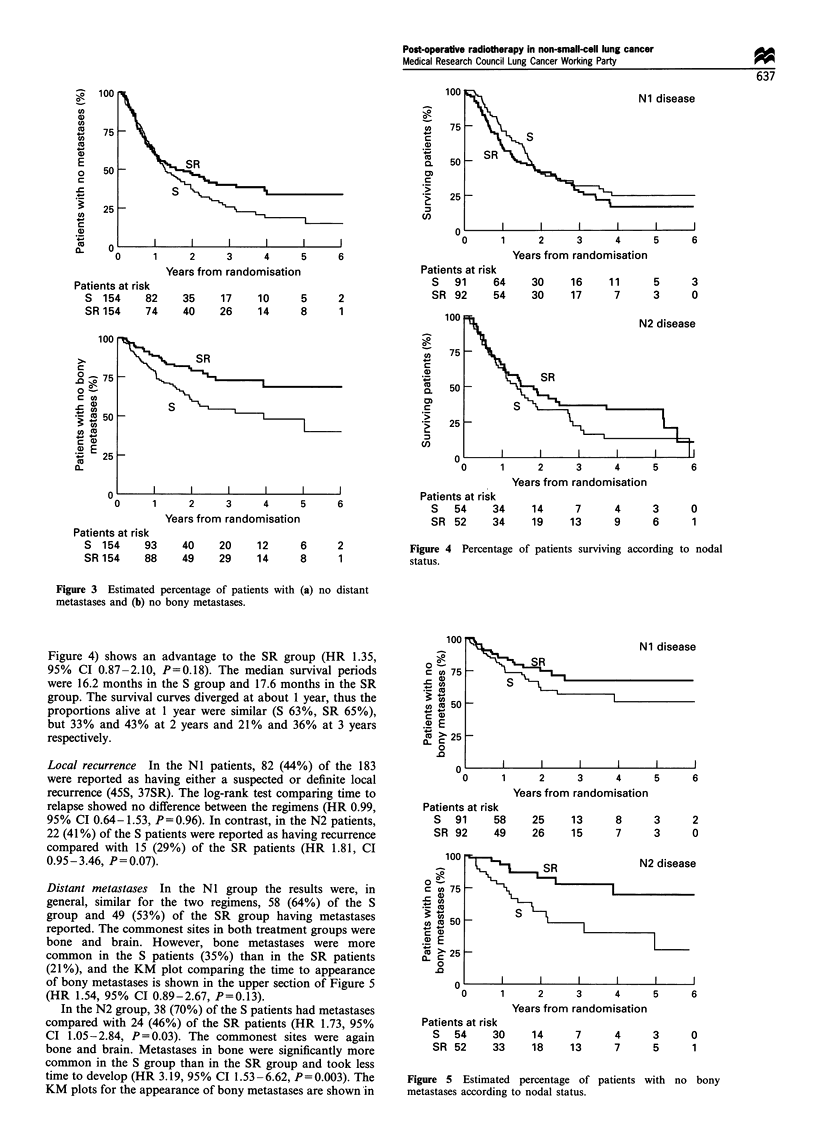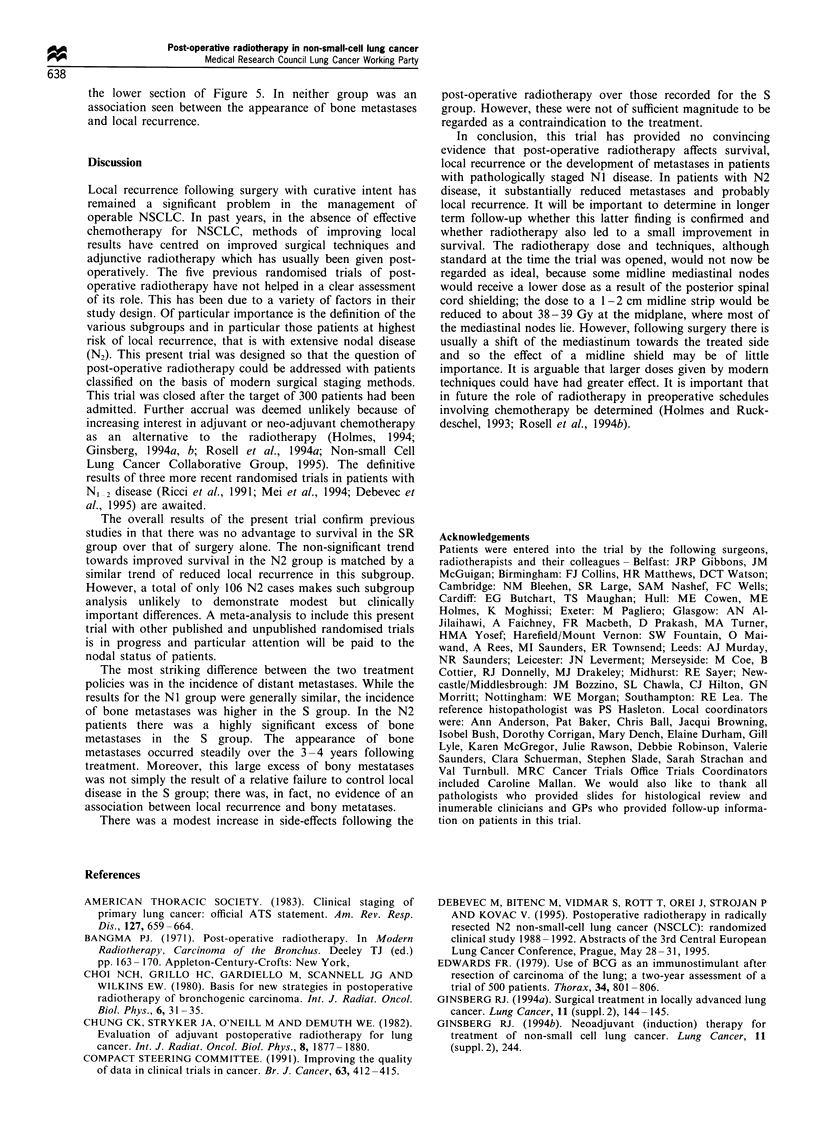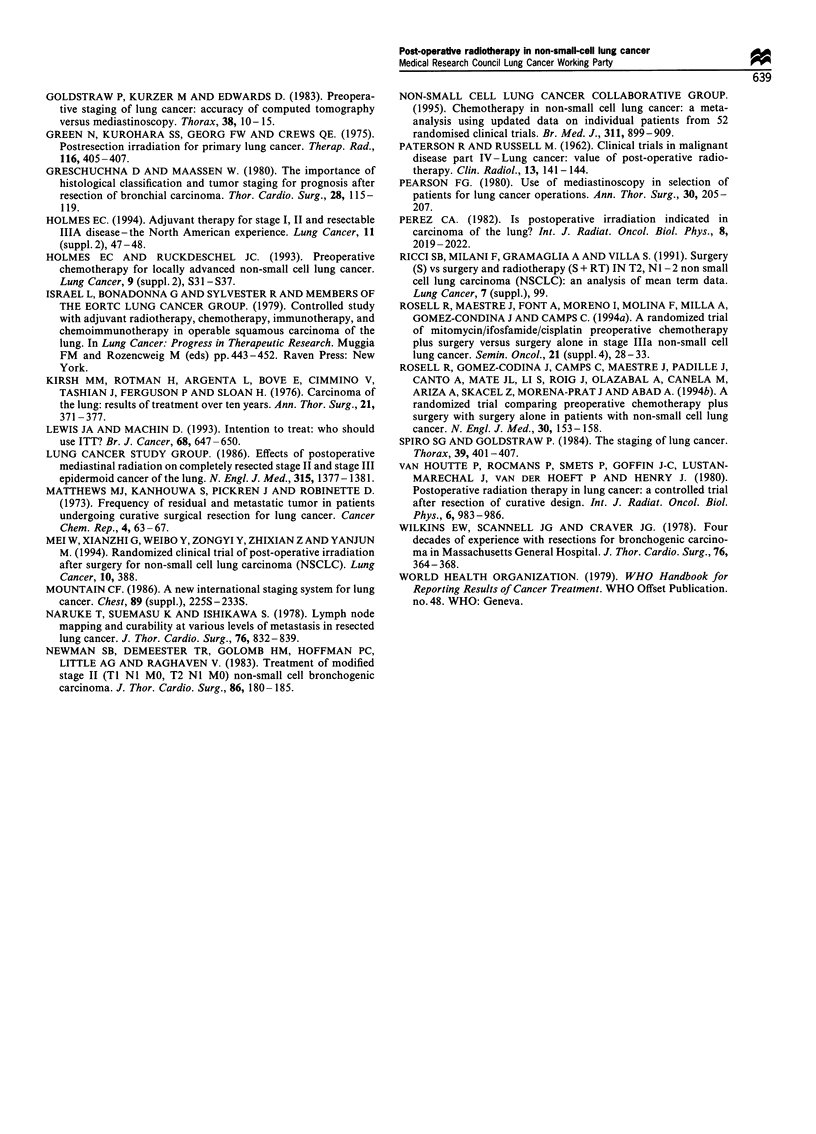# The role of post-operative radiotherapy in non-small-cell lung cancer: a multicentre randomised trial in patients with pathologically staged T1-2, N1-2, M0 disease. Medical Research Council Lung Cancer Working Party.

**DOI:** 10.1038/bjc.1996.413

**Published:** 1996-08

**Authors:** R. J. Stephens, D. J. Girling, N. M. Bleehen, K. Moghissi, H. M. Yosef, D. Machin

**Affiliations:** MRC Cancer Trials Office Cambridge, UK.

## Abstract

The role of post-operative radiotherapy for patients with non-small-cell lung cancer (NSCLC) is unclear despite five previous randomised trials. One deficiency with these trials was that they did not include adequate TNM staging, and so the present randomised trial was designed to compare surgery alone (S) with surgery plus post-operative radiotherapy (SR) in patients with pathologically staged T1-2, N1-2. M0 NSCLC. Between July 1986 and October 1993, 308 patients (154 S, 154 SR) were entered from 16 centres in the UK. The median age of the patients was 62 years, 74% were male, > 85% had normal or near normal levels of general condition, activity and breathlessness, 68% had squamous carcinoma, 52% had had a pneumonectomy, 63% had N1 disease and 37% N2 disease. SR patients received 40 Gy in 15 fractions starting 4-6 weeks post-operatively. Overall there was no advantage to either group in terms of survival, although definite local recurrence and bony metastases appeared less frequently and later in the SR group. In a subgroup analysis, in the N1 group no differences between the treatment groups were seen, but in the N2 group SR patients appeared to gain a one month survival advantage, delayed time to local recurrence and time to appearance of the bone metastases. There is, therefore, no clear indication for post-operative radiotherapy in N1 disease, but the question remains unresolved in N2 disease.


British Journal of Cancer (1996) 74, 632-639
© 1996 Stockton Press All rights reserved 0007-0920/96 $12.00

### The role of post-operative radiotherapy in non-small-cell lung cancer: a multicentre randomised trial in patients with pathologically staged $T_{1-2}$, $N_{1-2}$, $M_0$ disease

Medical Research Council Lung Cancer Working Party*
Prepared on behalf of the working party and its collaborators by RJ Stephens[1], DJ Girling[1], NM Bleehen[2], K Moghissi[3], HMA Yosef[4] and D Machin[1]

[1]*MRC Cancer Trials Office, 5 Shaftesbury Road, Cambridge CB2 2BW, UK;* [2]*MRC Clinical Oncology and Radiotherapeutics Unit, Addenbrooke's Hospital, Hills Road, Cambridge CB2 2QQ, UK;* [3]*Department of Cardiothoracic Surgery, Goole and District Hospital, Woodland Avenue, Goole, North Humberside DN14 6RX, UK;* [4]*Beatson Oncology Centre, Western Infirmary, Glasgow G11 6NT, UK.*

**Summary** The role of post-operative radiotherapy for patients with non-small-cell lung cancer (NSCLC) is unclear despite five previous randomised trials. One deficiency with these trials was that they did not include adequate TNM staging, and so the present randomised trial was designed to compare surgery alone (S) with surgery plus post-operative radiotherapy (SR) in patients with pathologically staged T1-2, N1-2, M0 NSCLC. Between July 1986 and October 1993, 308 patients (154 S, 154 SR) were entered from 16 centres in the UK. The median age of the patients was 62 years, 74% were male, >85% had normal or near normal levels of general condition, activity and breathlessness, 68% had squamous carcinoma, 52% had had a pneumonectomy, 63% had N1 disease and 37% N2 disease. SR patients received 40 Gy in 15 fractions starting 4-6 weeks post-operatively. Overall there was no advantage to either group in terms of survival, although definite local recurrence and bony metastases appeared less frequently and later in the SR group. In a subgroup analysis, in the N1 group no differences between the treatment groups were seen, but in the N2 group SR patients appeared to gain a one month survival advantage, delayed time to local recurrence and time to appearance of the bone metastases. There is, therefore, no clear indication for post-operative radiotherapy in N1 disease, but the question remains unresolved in N2 disease.

**Keywords:** non-small-cell lung cancer; randomised trial; post-operative radiotherapy

For patients with potentially resectable, non-small-cell lung cancer (NSCLC) without distant metastases, the standard treatment is an intended curative resection. In 1986, at the time this trial was planned and activated, a number of studies had already clarified some of the factors affecting resectability and subsequent prognosis.

Firstly, it was already clear that the duration of subsequent survival was greatly reduced if the mediastinal nodes were found to be involved. For example, in a consecutive series of 245 patients undergoing curative resection, Wilkins *et al.* (1978) reported 5-year survival rates of 42% in patients without mediastinal node involvement compared with only 16% with such involvement. Greschuchna and Maassen (1980) reported corresponding rates of 37% and 11%, and broadly similar results were reported by others (Edwards, 1979; Mountain, 1986).

Secondly, the importance of careful preoperative staging in the selection of patients suitable for intended resection was appreciated, particularly the need to avoid inappropriate thoracotomy in patients with T3 tumours, gross mediastinal node involvement or metastatic disease (Pearson, 1980; Spiro and Goldstraw, 1984). This need can only be met if, in patients otherwise suitable for thoracotomy, the mediastinum is staged, using such techniques as computerised tomography (CT), cervical mediastinoscopy and anterior mediastinotomy (if so indicated for left upper lobe tumours), and if clinical and laboratory indications of possible metastatic disease are adequately investigated (Matthews *et al.*, 1973; Goldstraw *et al.*, 1983; Spiro and Goldstraw, 1984).

Finally, in patients found to have resectable, potentially curable disease at thoracotomy, prognosis was known to be affected by whether the mediastinal nodes are found, at that stage, to be macroscopically or microscopically involved and, by which particular nodal groups are affected, emphasising the need, in assessing prognosis, for careful nodal mapping at thoracotomy (Naruke *et al.*, 1978; American Thoracic Society, 1983). Mediastinal nodes need to be sampled and labelled from the lung hilum, the main carinal group, and one other adjacent station, and their microscopical involvement reported separately.

In the light of the above observations, the question arises whether, among patients adequately assessed for resection, post-operative radiotherapy prolongs survival in those with, and in those without, mediastinal node involvement (Perez, 1982). A number of retrospective surveys claimed to show that it was of benefit to patients with mediastinal node metastases (Green *et al.*, 1975; Kirsh *et al.*, 1976; Choi *et al.*, 1980; Chung *et al.*, 1982; Newman *et al.*, 1983); but these surveys were in general small and involved unreliable comparsions against either historical controls or other series from different centres.

Results from five randomised trials, however, were inconclusive and inconsistent. Paterson and Russell (1962), in a trial of 202 patients randomised to pneumonectomy with or without post-operative mediastinal orthovoltage radiotherapy, showed no difference in survival, the 3-year survival rates being 36% in the no radiotherapy group and 33% in the radiotherapy group. However, the trial included 43 patients with poorly differentiated anaplastic or oat cell carcinoma, no TNM staging was done either before or during surgery, and the intake was stratified only by operating

Correspondence: RJ Stephens
*Members: NM Bleehen, JJ Bolger, PI Clark, K Connolly, DJ Girling, PS Hasleton, P Hopwood, FR Macbeth, D Machin, K Moghissi, MI Saunders, RJ Stephens, N Thatcher and RJ White
Received 23 January 1995; revised 22 January 1996; accepted 25 January 1996

surgeon and patient's age; the influence on the result of histology, mediastinal node involvement and other variables of possible prognostic importance is uncertain. A study by Bangma (1971) is often mentioned in this context, but it was not a randomised trial; the patients were assigned alternatively to the two treatment groups. Even so, in this small study (73 patients) also, no benefit was shown for post-operative radiotherapy.

In an analysis involving 175 of 224 randomised patients with tumour confined to the lung, that is, with no intra-operative evidence of lymph node metastases (Van Houtte *et al.*, 1980), survival was better in the non-irradiated group, the 5-year survival rate being 43% in this group compared with 24% in the irradiated group. Although the survival difference was not statistically significant, the size of the effect observed raised the question whether post-operative mediastinal radiotherapy might be harmful in patients with N0 or N1 disease.

In a trial by the Lung Cancer Study Group (1986), in which 230 patients with resected stage II or III M0, squamous carcinomas were pathologically staged intra-operatively and in which the randomisation was stratified by stage, weight loss, age and institution, there was no evidence that post-operative radiotherapy improved survival, even in patients with N2 disease, although in this latter group the local recurrence rate was substantially and significantly lower.

In contrast to the above four trials, in a randomised trial conducted by the European Organization for Research and Treatment of Cancer, 230 of 392 patients with squamous carcinoma were evaluated, 88 of whom had regional lymph node metastases (Israel *et al.*, 1979). The 3-year tumour-free survival rate was 70% in 104 patients given post-resection radiotherapy compared with 50% in 126 treated by resection without post-operative radiotherapy. This result must be treated with caution because of the large and unbalanced numbers of patients excluded, and full details have not been published in a refereed journal.

The present trial was therefore undertaken to make a randomised comparison of post-operative mediastinal radiotherapy *vs* no radiotherapy in patients with NSCLC pathologically staged as T1–2, N1–2, M0. Although patients would be randomised after a resection that was considered complete and potentially curative, they would be carefully staged to ensure entering only a minimum of those with unsuspected metastatic disease. The two treatment policies were to be compared in terms of survival, time to local recurrence, site and time to occurrence of metastases, and general condition, performance status and breathlessness. However, in view of the results, summarised above, of the other randomised trials, it was considered that the addition of post-operative radiotherapy might be less effective in patients with N1 disease compared with those with microscopic N2 disease, or might even be deleterious in patients with N1 disease (Van Houtte *et al.*, 1980). It was therefore planned that, within this one trial, as well as obtaining an overall estimate of treatment effect, a subgroup analysis for the two nodal status groups should be presented.

### Materials and methods

### *Preoperative eligibility criteria*

Patients of either sex aged 75 years or less were potentially eligible for the trial if preoperatively they had normal or near normal activity (WHO grades 0–2, World Health Organization, 1979), even if symptoms were present. They had to have lung and cardiac function adequate for the proposed resection, to have received no previous radiotherapy or chemotherapy and no previous surgery (except diagnostic) for the current disease, and to have no other concomitant malignant disease and no other serious condition contra-indicating surgery or radiotherapy.

Preoperative staging was recommended, using wherever possible a CT scan of the chest (including mediastinum and contralateral lung), upper abdomen (including liver, adrenals and kidneys) and brain. If the CT chest scan showed mediastinal abnormalities, cervical mediastinoscopy and (for left upper lobe tumours) anterior mediastinotomy were recommended. If CT scans could not be performed, mediastinoscopy or mediastinotomy were to be done whenever possible. If there was radiographic or bronchoscopic evidence of mediastinal lymphadenopathy, CT scan, mediastinoscopy or mediastinotomy were recommended. If there was any reason to suspect metastases it was required that every effort be made to confirm or exclude them by whatever additional investigations were deemed necessary.

### *Operative and post-operative eligibility criteria*

Patients had to have NSCLC of any cell type, except carcinoid tumour, which was pathologically staged as T1–2, N1–2, M0. Histological diagnosis and the pathological T staging, based on the resected specimen, were made by the local histopathologist. To ensure uniformity of classification, histology slides were reviewed by a single reference histopathologist.

Patients must have had an intended curative resection deemed to have been complete. Patients were ineligible if the bronchial margins of the resection specimen were invaded by cancer on local histopathological examination, but those with the visceral pleura involved were eligible provided the tumour was not adherent to the parietal pleura and there was no pleural effusion. Patients should have had no evidence of residual or metastatic disease during the first 2 weeks post-operatively.

At operation as many as possible of the ipsilateral lymph node stations (Naruke *et al.*, 1978) were to be sampled, including at least two mediastinal stations, the main subcarinal nodes and (except for lower lobe tumours) the high paratracheal nodes. For lower lobe tumours, the para-oesophageal or pulmonary ligament nodes were to be sampled. The pathological N staging was made by the local histopathologist.

### *Treatment allocation*

Once the trial had been approved by the local ethics committee and individual patient consent was obtained, clinicians telephoned the MRC Cancer Trials Office between 2 and 4 weeks post-operatively to obtain a treatment allocation to either radiotherapy or no radiotherapy. Patients were allocated to one of the two regimens using a minimisation procedure stratifying for surgeon, TNM stage (T1N1M0, T2N1M0, T1N2M0, T2N2M0 or unknown at time of randomisation) and histology (squamous, adenocarcinoma, large cell, other or unknown at time of randomisation).

### *Surgery with no post-operative radiotherapy (S)*

Patients in this group received no further specific anti-cancer treatment unless their disease recurred. Any subsequent treatment was given at the discretion of the individual clinician.

### *Surgery with post-operative radiotherapy (SR)*

Patients allocated to immediate post-operative radiotherapy received a course of radiotherapy to include the bronchial stump, mediastinal nodes and both lung hila, given in accordance with local practice, starting 4 to 6 weeks after the date of operation. If the tumour was in an upper lobe or if high mediastinal nodes were involved, the field included both supraclavicular fossae. The upper border of the field was never lower than the supraclavicular notch and the lower border extended at least 3 cm below the carina. The central midline dose was 40 Gy given in daily fractions (f) 5 days a week over 3 weeks using megavoltage x-ray therapy or $^{60}Co$ gamma-ray teletherapy. Parallel opposed fields were to be used with no correction for extra transmission through lung tissue. The spinal cord dose was limited to 35 Gy by the use of posterior lead blocks. Because of the greater contribution from the anterior field at the midplane than at the cord, the midplane dose was likely to be above 35 Gy.

*Reports and investigations*

The admission report included details of the staging procedures, measurement of the blood haemoglobin, white cell and platelet counts, an assessment of general condition, WHO performance status and degree of breathlessness according to the groupings shown in Table II, details of the operation (extent of resection and lymph node station numbers sampled), site of tumour, any post-operative complications experienced and the TNM stage.

Patients in the S group were seen and assessed at 6 and 9 weeks from operation, and those in the SR group at the start and end of radiotherapy. Both groups were then seen at 4, 6, 9, 12, 18 and 24 months from operation, and annually thereafter. The follow-up reports included details of the patient's blood counts, any evidence of recurrence of the primary cancer, metastases and any anti-cancer treatment. Clinicians reported the presence of recurrence and metastases as 'definite' or 'suspected' according to whatever evidence was available to them. They also reported the patients' general condition, WHO performance status and degree of breathlessness.

All patients were followed to death, even if they were subsequently found not to have satisfied the eligibility criteria for the trial, or if they did not for any reason receive the allocated treatment.

*Statistical methods*

All analyses are based on the intention to treat principle (Lewis and Machin, 1993). Survival was calculated from the date of randomisation until death or the date last known to be alive. The Kaplan-Meier (KM) estimate was used to calculate survival curves and the Mantel-Cox version of the log-rank test to make treatment comparisons. Associated confidence intervals (CIs) for the corresponding hazard ratios (HRs) were calculated. The same techniques were used to estimate and compare the time to occurrence of local recurrence and distant metastases. Patients with no such reported event were censored at date of death or date last seen alive.

The trial data were managed using the COMPACT program (COMPACT Steering Committee, 1991).

The proposed intake of 300 patients was based on the calculation that 5-year survival rates of 20% (S group) and 36% (SR group) would provide a 90% chance of obtaining a significant result at the 5% level. It was recognised that these figures were approximate because of the unpredictable mix of patients with N1 and N2 disease. It was hoped that this intake would be accrued within 3 years.

### Results

*Patients in the trial*

Between July 1986 and October 1993 308 patients were admitted from 16 centres in the UK. Exactly half (154) were allocated to each treatment.

*Preoperative characteristics*

Table I shows the staging procedures used and the numbers considered abnormal, suspicious and normal. The majority of patients had chest radiography (97%) and a bronchoscopy (95%) and of these 94% and 72%, respectively, were abnormal. A total of 73% of the patients had a CT chest scan and 95% of these were considered abnormal. Only a small proportion of patients had cervical mediastinoscopy (8%), anterior mediastinoscopy (3%) and mediastinotomy (2%).

Most patients (229, 74%) also had additional staging procedures to exlcude the presence of metastases. Table I shows that 38% of patients had a CT of the upper abdomen and 39% an abdominal ultrasound (five patients having both). The other investigations suggested in the protocol were very rarely used. Using these additional staging procedures, abnormalities were observed in five patients (3S, 2SR), but none of these were considered to be caused by metastases.

Nearly all the patients (Table II) had normal or near normal categories (grades 0 or 1) of general condition (90%), performance status (97%) and breathlessness (85%). The majority were male (74%), and at operation the median age was 62 years (range 37-77 years). No patients had an abnormally low ($<3000$ mm$^{-3}$) white blood cell count or low platelet count ($<100000$ mm$^{-3}$), but six patients (2S, 4SR) had a haemoglobin of $<10.0$ g dl$^{-1}$ and three (2S, 1SR) a high white cell count of $>20000$ mm$^{-3}$.

*Operative and post-operative characteristics*

Fifty-two per cent of the patients (Table III) had had a pneumonectomy (48% of the patients with N1 disease, 58% of those with N2) and 45% a lobectomy (48% of those with N1, 41% of those with N2). The majority had squamous carcinoma (68%) and the site of disease was left upper lobe in 36%, right upper lobe in 22%, left lower lobe in 15% and right lower lobe in 13%.

The median number of node groups sampled was three (range 0-9), and of these a median of one was found to be involved. The majority (85%) of patients in whom the hilar nodes were the most central group involved were classified as having N1 disease.

**Table I** Preoperative staging procedures

| *Staging procedures* | *S* No. (%) | *SR* No. (%) | *Total* No. (%) |
|---|---|---|---|
| To confirm disease[a] | | | |
| Chest radiography | | | |
| Abnormal | 143 (95) | 136 (92) | 279 (94) |
| Suspicious | 5 (3) | 9 (6) | 14 (5) |
| Normal | 2 (1) | 3 (2) | 5 (2) |
| NA/ not done | 4 | 6 | 10 |
| Bronchoscopy | | | |
| Abnormal | 105 (72) | 108 (72) | 213 (72) |
| Suspicious | 8 (6) | 14 (9) | 22 (7) |
| Normal | 32 (22) | 27 (18) | 59 (20) |
| NA/ not done | 9 | 5 | 14 |
| CT scan of chest | | | |
| Abnormal | 113 (97) | 101 (93) | 214 (95) |
| Suspicious | 3 (3) | 7 (6) | 10 (4) |
| Normal | 0 (0) | 1 (1) | 1 (0) |
| NA/ not done | 38 | 45 | 83 |
| To exclude distant metastases[b] | | | |
| CT upper abdomen | | | |
| Normal | 64 (100) | 50 (93) | 114 (97) |
| Suspicious | 0 (0) | 3 (6) | 3 (3) |
| Normal | 0 (0) | 1 (2) | 1 (1) |
| NA/ not done | 90 | 100 | 190 |
| Abdominal ultrasound | | | |
| Abnormal | 54 (100) | 64 (97) | 118 (98) |
| Suspicious | 0 (0) | 1 (2) | 1 (1) |
| Abnormal | 0 (0) | 1 (2) | 1 (1) |
| NA/ not done | 100 | 88 | 188 |
| Total patients | 154 | 154 | 308 |

[a]24 patients had cervical mediastinoscopy, 10 anterior mediastinoscopy, 5 mediastinotomy and 0 gallium chest scan. [b]5 patients had a CT brain scan (1 S abnormal), 2 a laparoscopy, 3 bone radiology (1 S abnormal), 36 radioisotope bone scan (1 S abnormal) and 1 a marrow trephine.

As reported over the telephone at the time of randomisation, 47% of the patients had T2N1M0 disease, 29% T2N2M0, 16% T1N1M0 and only 8% T1N2M0.

The median time from operation to randomisation was 15 days (range 3–167 days), 133 (43%) being earlier and 41 (13%) later than the protocol recommendation of 14–28 days.

The distributions of all the above characteristic between the two regimens were very similar.

*Ineligible patients*

Seventeen patients (10S, 7SR) did not fit the exact eligibility criteria as laid down in the protocol: two (both S) had no evidence of lymph node involvement, two (both SR) had bronchial margins involved, one (S) had lung metastases, one (SR) had had previous radiotherapy for breast cancer, four (2S, 2SR) were aged >75 years and seven (5S, 2SR) were randomised more than 8 weeks after their operation. However, all 17 patients have been included in all the following analyses which are based on the intention to treat principle. An analysis excluding these patients made no material difference to the conclusions.

*Protocol treatment received*

Of the 154 SR patients, 15 (10%) did not start radiotherapy and 3 (2%) did not complete their allocated course. Of the 15 who did not start this was because of refusal in four patients, death in three, severe illness in three, administrative error in one, previous radiotherapy for breast cancer in one and refusal by the radiotherapist to give the protocol regimen in one. For the remaining two, no reason was given. Of the three patients who did not complete their full course (40 Gy/15 f): one refused after 5 f, one stopped after 12 f because of brain metastases and one stopped after 13 f because of severe oesophagitis.

Of the 136 patients who did receive their allocated course of post-operative radiotherapy, 55 (36% of the 154 patients allocated) had radiotherapy given exactly as specified in the protocol, and 21 patients (14%) had minor deviations, defined as being within 10% of the protocol specification. A further 57 patients (37%) had the correct treatment, or within 10% of it (in terms of dose and fractions), but deviated from the protocol in the timing, field size or number of fields. Of the remaining three patients (2%), two received 40 Gy in 20 f and one received 32 Gy in 8 f.

*Adverse effects*

*Post-operative complications* In these patients, who all had to have survived a successful resection for a minimum of 2 weeks post-surgery to be eligible for the trial, few post-operative complications were reported: 14 patients had mild and 7 moderate lung infections, three mild and one moderate wound infections, and one mild and one moderate pleural infections. In addition, 32 patients had some other minor complications.

*Reactions to allocated radiotherapy* In contrast however a large proportion, 96 (69%) of the 139 patients who were allocated to and started radiotherapy were reported as having some adverse reactions to it. The main reactions mentioned were oesophagitis, dysphagia, nausea, vomiting, sore throat and lethargy. In 36 patients, symptoms were considered mild, in 45 moderate and in 15 severe.

*Further treatment*

The numbers of patients requiring further surgery or treatment with chemotherapy or radical radiotherapy at any time were very similar in the two regimens. Ten patients in the S group had further surgery as did ten in the SR group, ten and five patients, respectively, received chemotherapy, and three S patients had radical thoracic radiotherapy. However, 44 (29%) patients in the S group received

**Table II** Preoperative characteristics

| *Characterisitc* | *S* No. (%) | *SR* No. (%) | *Total* No. (%) |
|---|---|---|---|
| Sex | | | |
| Male | 117 (76) | 112 (73) | 229 (74) |
| Female | 37 (24) | 42 (27) | 79 (26) |
| Age | | | |
| <45 | 5 (3) | 2 (1) | 7 (2) |
| 45–54 | 18 (12) | 26 (17) | 44 (14) |
| 55–64 | 70 (45) | 70 (45) | 140 (45) |
| 65–74 | 58 (38) | 53 (34) | 111 (36) |
| 75+ | 3 (2) | 3 (2) | 6 (2) |
| General condition | | | |
| 0 Excellent | 35 (23) | 38 (25) | 73 (24) |
| 1 Good | 104 (68) | 99 (65) | 203 (66) |
| 2 Fair | 14 (9) | 16 (10) | 30 (10) |
| NA | 1 | 1 | 2 |
| WHO performance status | | | |
| 0 Normal | 64 (44) | 71 (48) | 135 (46) |
| 1 Activity restricted | 77 (53) | 70 (48) | 147 (50) |
| 2 Up≥50% waking time | 4 (3) | 6 (4) | 10 (3) |
| NA | 9 | 7 | 16 |
| Breathlessness | | | |
| 0 No dyspnoea | 61 (41) | 63 (42) | 124 (42) |
| 1 Walks on flat | 65 (44) | 64 (42) | 129 (43) |
| 2 Walks over 100 yards | 21 (14) | 24 (16) | 45 (15) |
| NA | 7 | 3 | 10 |
| Total patients | 154 | 154 | 308 |

**Table III** Operative and post-operative characteristics

| *Characterisitc* | *S* No. (%) | *SR* No. (%) | *Total* No. (%) |
|---|---|---|---|
| Operation | | | |
| Pneumonectomy | 75 (49) | 84 (55) | 159 (52) |
| Lobectomy | 76 (49) | 64 (42) | 140 (45) |
| Segmentectomy | 3 (2) | 4 (3) | 7 (2) |
| Sleeve resection | 0 (0) | 2 (1) | 2 (1) |
| Histology[a] | | | |
| Squamous | 101 (67) | 101 (68) | 202 (68) |
| Adenocarcinoma | 36 (24) | 36 (24) | 72 (24) |
| Large cell | 10 (7) | 8 (5) | 18 (6) |
| Other | 3 (2) | 4 (3) | 7 (2) |
| Not reported | 4 | 5 | 9 |
| Site | | | |
| Left | | | |
| Upper Lobe | 55 (36) | 55 (36) | 110 (36) |
| Hilum | 7 (5) | 9 (6) | 16 (5) |
| Lower Lobe | 24 (16) | 21 (14) | 45 (15) |
| Indeterminate | 0 (0) | 1 (1) | 1 (0) |
| Right | | | |
| Upper Lobe | 38 (25) | 30 (19) | 68 (22) |
| Hilum | 2 (1) | 7 (5) | 9 (3) |
| Middle Lobe | 5 (3) | 10 (6) | 15 (5) |
| Lower Lobe | 21 (14) | 20 (13) | 41 (13) |
| Indeterminate | 1 (1) | 1 (1) | 2 (1) |
| Not reported | 1 | 0 | 1 |
| TNM stage[a] | | | |
| T1 N1 M0 | 23 (16) | 23 (16) | 46 (16) |
| T2 N1 M0 | 68 (47) | 69 (48) | 137 (47) |
| T1 N2 M0 | 13 (9) | 10 (7) | 23 (8) |
| T2 N2 M0 | 41 (28) | 42 (29) | 83 (29) |
| Not reported | 9 | 10 | 19 |
| Total patients | 154 | 154 | 308 |

[a]Stratification factor.

palliative radiotherapy compared with 28 (18%) in the SR group. Most of this (74%) was for metastases.

*Survival*

Of the 308 patients, 221 (72%) have died, and the median follow-up of the 87 surviving patients is 30 months, range 7 months to 7 years. Figure 1 shows the KM plots by treatment regimen. Although initially there appears to be a slight advantage to the S group, and the median survival times are 19 months for the S group and 17.5 months for the SR group, the curves cross at approximately 20 months and the HR is 1.00 (95% CI 0.77–1.30, $P$=0.99).

*Local recurrence*

Local recurrence was reported by the clinicians as none, suspected or definite. A total of 72 (23%) patients (45S, 27SR) were reported as having definite, and a further 59 (19%) patients (28S, 31SR) as having suspected local recurrence at some time in their lives. The time to the first reporting of recurrence (either suspected or definite) was plotted using KM curves, patients who died without local recurrence being censored at the date of death. The log-rank plots are shown in Figure 2. There was no clear evidence that SR was better than S (HR 1.23, 95% CI 0.87–1.73, $P$=0.24). However, an analysis of the time to *definite* local recurrence indicated an advantage to the SR regimen (HR 1.64, 95% CI 1.03–2.61, $P$=0.04).

| Patients at risk | | | | | | |
|---|---|---|---|---|---|---|
| S 154 | 104 | 47 | 24 | 16 | 8 | 3 |
| SR 154 | 94 | 54 | 32 | 17 | 9 | 2 |

**Figure 1** Percentage of patients surviving from date of randomisation.

| Patients at risk | | | | | | |
|---|---|---|---|---|---|---|
| S 154 | 88 | 31 | 16 | 10 | 5 | 2 |
| SR 154 | 72 | 38 | 23 | 14 | 8 | 1 |

**Figure 2** Estimated percentage of patients with no local recurrence.

*Distant metastases*

Table IV shows the number of patients in whom distant metastases were suspected or confirmed. The patterns of failure were very similar for the two regimens. Overall, distant metastases developed in 67% of the S group compared with 51% of the SR group, and the major sites were bone and brain. The log-rank test was used to check if there were differences in the time to occurrence. The upper section of Figure 3 shows the KM plots for the appearance of any metastases. Although the curves diverge at approximately 1 year, there was no overall difference (HR 1.28, 95% CI 0.95–1.71, $P$=0.10). The only specific site in which a significant difference was observed was bone (lower section of Figure 3), and in the S group bony metastases were more common and developed earlier (HR 2.09, 95% CI 1.35–3.22, $P$=0.001). Analyses using only confirmed metastases gave very similar results.

*Changes in general condition, performance status and breathlessness*

Comparing the percentages of patients with impaired (grade 2, 3 or 4) general condition, performance status and breathlessness at each of the nine assessments up to 2 years showed, for all three variables, that patients allocated to SR had a period of worse condition during and just after their radiotherapy (month 1), but thereafter no differences between the two regimens were observed (data not shown).

*Subgroup analysis of the N1 and N2 groups*

The TNM stage was reported over the telephone before randomisation, 183 patients being considered to have N1 disease (91 being allocated to the S group, 92 to the SR group), 106 N2 disease (54S, 52SR) and 19 unknown (Table III). The TNM stage was also recorded on the admission form but these data have not been used to replace or update the information, as the numbers of changes and the pattern of staging of those patients previously unknown, were different between the two treatment regimens. The following analyses are therefore based on 289 patients whose nodal status data were obtained prerandomisation (183 N1, 106 N2). However, repeat analyses using TNM stage from the admission form gave similar results.

*Survival* In the N1 group 126 (69%) patients have died (60 S, 66 SR) and the KM plot (upper section of Figure 4) suggests a slight advantage to the S group: the median survival was 20.5 months in the S group compared with 16.3 months in the SR group, and the proportions of patients surviving at 1 year were 71% and 60% respectively. However, by 2 years the curves had come together and there was no significant difference between the regimens (HR 0.82, 95% CI 0.58–1.16, $P$=0.26).

In the N2 group 43 (80%) of the S patients and 36 (69%) of the SR patients have died. The KM plot (lower section of

**Table IV** Occurrence of suspected or confirmed distant metastases

| *Metastases* | *S* No. (%) | *SR* No. (%) | *Log-rank* P-*value* |
|---|---|---|---|
| Any | 103 (67) | 79 (51) | 0.10 |
| Liver | 12 (8) | 15 (10) | 0.48 |
| Brain | 27 (18) | 28 (18) | 0.81 |
| Bone | 55 (36) | 27 (18) | 0.001 |
| Opposite lung | 21 (14) | 14 (9) | 0.26 |
| Nodes | 18 (12) | 17 (11) | 0.97 |
| Others | 19 (12) | 12 (8) | 0.35 |
| Total patients | 154 | 154 | |

Patients at risk

| | | | | | | | |
|---|---|---|---|---|---|---|---|
| S | 154 | 82 | 35 | 17 | 10 | 5 | 2 |
| SR | 154 | 74 | 40 | 26 | 14 | 8 | 1 |

Patients at risk

| | | | | | | | |
|---|---|---|---|---|---|---|---|
| S | 154 | 93 | 40 | 20 | 12 | 6 | 2 |
| SR | 154 | 88 | 49 | 29 | 14 | 8 | 1 |

**Figure 3** Estimated percentage of patients with (**a**) no distant metastases and (**b**) no bony metastases.

Figure 4) shows an advantage to the SR group (HR 1.35, 95% CI 0.87–2.10, $P=0.18$). The median survival periods were 16.2 months in the S group and 17.6 months in the SR group. The survival curves diverged at about 1 year, thus the proportions alive at 1 year were similar (S 63%, SR 65%), but 33% and 43% at 2 years and 21% and 36% at 3 years respectively.

*Local recurrence* In the N1 patients, 82 (44%) of the 183 were reported as having either a suspected or definite local recurrence (45S, 37SR). The log-rank test comparing time to relapse showed no difference between the regimens (HR 0.99, 95% CI 0.64–1.53, $P=0.96$). In contrast, in the N2 patients, 22 (41%) of the S patients were reported as having recurrence compared with 15 (29%) of the SR patients (HR 1.81, CI 0.95–3.46, $P=0.07$).

*Distant metastases* In the N1 group the results were, in general, similar for the two regimens, 58 (64%) of the S group and 49 (53%) of the SR group having metastases reported. The commonest sites in both treatment groups were bone and brain. However, bone metastases were more common in the S patients (35%) than in the SR patients (21%), and the KM plot comparing the time to appearance of bony metastases is shown in the upper section of Figure 5 (HR 1.54, 95% CI 0.89–2.67, $P=0.13$).

In the N2 group, 38 (70%) of the S patients had metastases compared with 24 (46%) of the SR patients (HR 1.73, 95% CI 1.05–2.84, $P=0.03$). The commonest sites were again bone and brain. Metastases in bone were significantly more common in the S group than in the SR group and took less time to develop (HR 3.19, 95% CI 1.53–6.62, $P=0.003$). The KM plots for the appearance of bony metastases are shown in

Patients at risk

| | | | | | | | |
|---|---|---|---|---|---|---|---|
| S | 91 | 64 | 30 | 16 | 11 | 5 | 3 |
| SR | 92 | 54 | 30 | 17 | 7 | 3 | 0 |

Patients at risk

| | | | | | | | |
|---|---|---|---|---|---|---|---|
| S | 54 | 34 | 14 | 7 | 4 | 3 | 0 |
| SR | 52 | 34 | 19 | 13 | 9 | 6 | 1 |

**Figure 4** Percentage of patients surviving according to nodal status.

Patients at risk

| | | | | | | | |
|---|---|---|---|---|---|---|---|
| S | 91 | 58 | 25 | 13 | 8 | 3 | 2 |
| SR | 92 | 49 | 26 | 15 | 7 | 3 | 0 |

Patients at risk

| | | | | | | | |
|---|---|---|---|---|---|---|---|
| S | 54 | 30 | 14 | 7 | 4 | 3 | 0 |
| SR | 52 | 33 | 18 | 13 | 7 | 5 | 1 |

**Figure 5** Estimated percentage of patients with no bony metastases according to nodal status.

the lower section of Figure 5. In neither group was an association seen between the appearance of bone metastases and local recurrence.

### Discussion

Local recurrence following surgery with curative intent has remained a significant problem in the management of operable NSCLC. In past years, in the absence of effective chemotherapy for NSCLC, methods of improving local results have centred on improved surgical techniques and adjunctive radiotherapy which has usually been given post-operatively. The five previous randomised trials of post-operative radiotherapy have not helped in a clear assessment of its role. This has been due to a variety of factors in their study design. Of particular importance is the definition of the various subgroups and in particular those patients at highest risk of local recurrence, that is with extensive nodal disease ($N_2$). This present trial was designed so that the question of post-operative radiotherapy could be addressed with patients classified on the basis of modern surgical staging methods. This trial was closed after the target of 300 patients had been admitted. Further accrual was deemed unlikely because of increasing interest in adjuvant or neo-adjuvant chemotherapy as an alternative to the radiotherapy (Holmes, 1994; Ginsberg, 1994*a*, *b*; Rosell *et al.*, 1994*a*; Non-small Cell Lung Cancer Collaborative Group, 1995). The definitive results of three more recent randomised trials in patients with $N_{1-2}$ disease (Ricci *et al.*, 1991; Mei *et al.*, 1994; Debevec *et al.*, 1995) are awaited.

The overall results of the present trial confirm previous studies in that there was no advantage to survival in the SR group over that of surgery alone. The non-significant trend towards improved survival in the N2 group is matched by a similar trend of reduced local recurrence in this subgroup. However, a total of only 106 N2 cases makes such subgroup analysis unlikely to demonstrate modest but clinically important differences. A meta-analysis to include this present trial with other published and unpublished randomised trials is in progress and particular attention will be paid to the nodal status of patients.

The most striking difference between the two treatment policies was in the incidence of distant metastases. While the results for the N1 group were generally similar, the incidence of bone metastases was higher in the S group. In the N2 patients there was a highly significant excess of bone metastases in the S group. The appearance of bone metastases occurred steadily over the 3–4 years following treatment. Moreover, this large excess of bony mestatases was not simply the result of a relative failure to control local disease in the S group; there was, in fact, no evidence of an association between local recurrence and bony metatases.

There was a modest increase in side-effects following the post-operative radiotherapy over those recorded for the S group. However, these were not of sufficient magnitude to be regarded as a contraindication to the treatment.

In conclusion, this trial has provided no convincing evidence that post-operative radiotherapy affects survival, local recurrence or the development of metastases in patients with pathologically staged N1 disease. In patients with N2 disease, it substantially reduced metastases and probably local recurrence. It will be important to determine in longer term follow-up whether this latter finding is confirmed and whether radiotherapy also led to a small improvement in survival. The radiotherapy dose and techniques, although standard at the time the trial was opened, would not now be regarded as ideal, because some midline mediastinal nodes would receive a lower dose as a result of the posterior spinal cord shielding; the dose to a 1–2 cm midline strip would be reduced to about 38–39 Gy at the midplane, where most of the mediastinal nodes lie. However, following surgery there is usually a shift of the mediastinum towards the treated side and so the effect of a midline shield may be of little importance. It is arguable that larger doses given by modern techniques could have had greater effect. It is important that in future the role of radiotherapy in preoperative schedules involving chemotherapy be determined (Holmes and Ruckdeschel, 1993; Rosell *et al.*, 1994*b*).

**Acknowledgements**
Patients were entered into the trial by the following surgeons, radiotherapists and their colleagues – Belfast: JRP Gibbons, JM McGuigan; Birmingham: FJ Collins, HR Matthews, DCT Watson; Cambridge: NM Bleehen, SR Large, SAM Nashef, FC Wells; Cardiff: EG Butchart, TS Maughan; Hull: ME Cowen, ME Holmes, K Moghissi; Exeter: M Pagliero; Glasgow: AN Al-Jilaihawi, A Faichney, FR Macbeth, D Prakash, MA Turner, HMA Yosef; Harefield/Mount Vernon: SW Fountain, O Maiwand, A Rees, MI Saunders, ER Townsend; Leeds: AJ Murday, NR Saunders; Leicester: JN Leverment; Merseyside: M Coe, B Cottier, RJ Donnelly, MJ Drakeley; Midhurst: RE Sayer; Newcastle/Middlesbrough: JM Bozzino, SL Chawla, CJ Hilton, GN Morritt; Nottingham: WE Morgan; Southampton: RE Lea. The reference histopathologist was PS Hasleton. Local coordinators were: Ann Anderson, Pat Baker, Chris Ball, Jacqui Browning, Isobel Bush, Dorothy Corrigan, Mary Dench, Elaine Durham, Gill Lyle, Karen McGregor, Julie Rawson, Debbie Robinson, Valerie Saunders, Clara Schuerman, Stephen Slade, Sarah Strachan and Val Turnbull. MRC Cancer Trials Office Trials Coordinators included Caroline Mallan. We would also like to thank all pathologists who provided slides for histological review and inumerable clinicians and GPs who provided follow-up information on patients in this trial.

### References

AMERICAN THORACIC SOCIETY. (1983). Clinical staging of primary lung cancer: official ATS statement. *Am. Rev. Resp. Dis.*, **127**, 659–664.

BANGMA PJ. (1971). Post-operative radiotherapy. In *Modern Radiotherapy, Carcinoma of the Bronchus*. Deeley TJ (ed.) pp. 163–170. Appleton-Century-Crofts: New York,

CHOI NCH, GRILLO HC, GARDIELLO M, SCANNELL JG AND WILKINS EW. (1980). Basis for new strategies in postoperative radiotherapy of bronchogenic carcinoma. *Int. J. Radiat. Oncol. Biol. Phys.*, **6**, 31–35.

CHUNG CK, STRYKER JA, O'NEILL M AND DEMUTH WE. (1982). Evaluation of adjuvant postoperative radiotherapy for lung cancer. *Int. J. Radiat. Oncol. Biol. Phys.*, **8**, 1877–1880.

COMPACT STEERING COMMITTEE. (1991). Improving the quality of data in clinical trials in cancer. *Br. J. Cancer*, **63**, 412–415.

DEBEVEC M, BITENC M, VIDMAR S, ROTT T, OREI J, STROJAN P AND KOVAC V. (1995). Postoperative radiotherapy in radically resected N2 non-small-cell lung cancer (NSCLC): randomized clinical study 1988–1992. Abstracts of the 3rd Central European Lung Cancer Conference, Prague, May 28–31, 1995.

EDWARDS FR. (1979). Use of BCG as an immunostimulant after resection of carcinoma of the lung; a two-year assessment of a trial of 500 patients. *Thorax*, **34**, 801–806.

GINSBERG RJ. (1994*a*). Surgical treatment in locally advanced lung cancer. *Lung Cancer*, **11** (suppl. 2), 144–145.

GINSBERG RJ. (1994*b*). Neoadjuvant (induction) therapy for treatment of non-small cell lung cancer. *Lung Cancer*, **11** (suppl. 2), 244.

GOLDSTRAW P, KURZER M AND EDWARDS D. (1983). Preoperative staging of lung cancer: accuracy of computed tomography versus mediastinoscopy. *Thorax*, **38**, 10–15.

GREEN N, KUROHARA SS, GEORG FW AND CREWS QE. (1975). Postresection irradiation for primary lung cancer. *Therap. Rad.*, **116**, 405–407.

GRESCHUCHNA D AND MAASSEN W. (1980). The importance of histological classification and tumor staging for prognosis after resection of bronchial carcinoma. *Thor. Cardio. Surg.*, **28**, 115–119.

HOLMES EC. (1994). Adjuvant therapy for stage I, II and resectable IIIA disease–the North American experience. *Lung Cancer*, **11** (suppl. 2), 47–48.

HOLMES EC AND RUCKDESCHEL JC. (1993). Preoperative chemotherapy for locally advanced non-small cell lung cancer. *Lung Cancer*, **9** (suppl. 2), S31–S37.

ISRAEL L, BONADONNA G AND SYLVESTER R AND MEMBERS OF THE EORTC LUNG CANCER GROUP. (1979). Controlled study with adjuvant radiotherapy, chemotherapy, immunotherapy, and chemoimmunotherapy in operable squamous carcinoma of the lung. In *Lung Cancer: Progress in Therapeutic Research.* Muggia FM and Rozencweig M (eds) pp. 443–452. Raven Press: New York.

KIRSH MM, ROTMAN H, ARGENTA L, BOVE E, CIMMINO V, TASHIAN J, FERGUSON P AND SLOAN H. (1976). Carcinoma of the lung: results of treatment over ten years. *Ann. Thor. Surg.*, **21**, 371–377.

LEWIS JA AND MACHIN D. (1993). Intention to treat: who should use ITT? *Br. J. Cancer*, **68**, 647–650.

LUNG CANCER STUDY GROUP. (1986). Effects of postoperative mediastinal radiation on completely resected stage II and stage III epidermoid cancer of the lung. *N. Engl. J. Med.*, **315**, 1377–1381.

MATTHEWS MJ, KANHOUWA S, PICKREN J AND ROBINETTE D. (1973). Frequency of residual and metastatic tumor in patients undergoing curative surgical resection for lung cancer. *Cancer Chem. Rep.*, **4**, 63–67.

MEI W, XIANZHI G, WEIBO Y, ZONGYI Y, ZHIXIAN Z AND YANJUN M. (1994). Randomized clinical trial of post-operative irradiation after surgery for non-small cell lung carcinoma (NSCLC). *Lung Cancer*, **10**, 388.

MOUNTAIN CF. (1986). A new international staging system for lung cancer. *Chest*, **89** (suppl.), 225S–233S.

NARUKE T, SUEMASU K AND ISHIKAWA S. (1978). Lymph node mapping and curability at various levels of metastasis in resected lung cancer. *J. Thor. Cardio. Surg.*, **76**, 832–839.

NEWMAN SB, DEMEESTER TR, GOLOMB HM, HOFFMAN PC, LITTLE AG AND RAGHAVEN V. (1983). Treatment of modified stage II (T1 N1 M0, T2 N1 M0) non-small cell bronchogenic carcinoma. *J. Thor. Cardio. Surg.*, **86**, 180–185.

NON-SMALL CELL LUNG CANCER COLLABORATIVE GROUP. (1995). Chemotherapy in non-small cell lung cancer: a meta-analysis using updated data on individual patients from 52 randomised clinical trials. *Br. Med. J.*, **311**, 899–909.

PATERSON R AND RUSSELL M. (1962). Clinical trials in malignant disease part IV–Lung cancer: value of post-operative radiotherapy. *Clin. Radiol.*, **13**, 141–144.

PEARSON FG. (1980). Use of mediastinoscopy in selection of patients for lung cancer operations. *Ann. Thor. Surg.*, **30**, 205–207.

PEREZ CA. (1982). Is postoperative irradiation indicated in carcinoma of the lung? *Int. J. Radiat. Oncol. Biol. Phys.*, **8**, 2019–2022.

RICCI SB, MILANI F, GRAMAGLIA A AND VILLA S. (1991). Surgery (S) vs surgery and radiotherapy (S + RT) IN T2, N1–2 non small cell lung carcinoma (NSCLC): an analysis of mean term data. *Lung Cancer*, **7** (suppl.), 99.

ROSELL R, MAESTRE J, FONT A, MORENO I, MOLINA F, MILLA A, GOMEZ-CONDINA J AND CAMPS C. (1994*a*). A randomized trial of mitomycin/ifosfamide/cisplatin preoperative chemotherapy plus surgery versus surgery alone in stage IIIa non-small cell lung cancer. *Semin. Oncol.*, **21** (suppl. 4), 28–33.

ROSELL R, GOMEZ-CODINA J, CAMPS C, MAESTRE J, PADILLE J, CANTO A, MATE JL, LI S, ROIG J, OLAZABAL A, CANELA M, ARIZA A, SKACEL Z, MORENA-PRAT J AND ABAD A. (1994*b*). A randomized trial comparing preoperative chemotherapy plus surgery with surgery alone in patients with non-small cell lung cancer. *N. Engl. J. Med.*, **30**, 153–158.

SPIRO SG AND GOLDSTRAW P. (1984). The staging of lung cancer. *Thorax*, **39**, 401–407.

VAN HOUTTE P, ROCMANS P, SMETS P, GOFFIN J-C, LUSTAN-MARECHAL J, VAN DER HOEFT P AND HENRY J. (1980). Postoperative radiation therapy in lung cancer: a controlled trial after resection of curative design. *Int. J. Radiat. Oncol. Biol. Phys.*, **6**, 983–986.

WILKINS EW, SCANNELL JG AND CRAVER JG. (1978). Four decades of experience with resections for bronchogenic carcinoma in Massachusetts General Hospital. *J. Thor. Cardio. Surg.*, **76**, 364–368.

WORLD HEALTH ORGANIZATION. (1979). *WHO Handbook for Reporting Results of Cancer Treatment.* WHO Offset Publication. no. 48. WHO: Geneva.